# The Interaction of CRM1 and the Nuclear Pore Protein Tpr

**DOI:** 10.1371/journal.pone.0093709

**Published:** 2014-04-10

**Authors:** Charles L. Zhao, Seyed Hanif Mahboobi, Ruhollah Moussavi-Baygi, Mohammad R. K. Mofrad

**Affiliations:** Molecular Cell Biomechanics Laboratory, Departments of Bioengineering and Mechanical Engineering, University of California, Berkeley, California, United States of America; George Washington University, United States of America

## Abstract

While much has been devoted to the study of transport mechanisms through the nuclear pore complex (NPC), the specifics of interactions and binding between export transport receptors and the NPC periphery have remained elusive. Recent work has demonstrated a binding interaction between the exportin CRM1 and the unstructured carboxylic tail of Tpr, on the nuclear basket. Strong evidence suggests that this interaction is vital to the functions of CRM1. Using molecular dynamics simulations and a newly refined method for determining binding regions, we have identified nine candidate binding sites on CRM1 for C-Tpr. These include two adjacent to RanGTP – from which one is blocked in the absence of RanGTP – and three next to the binding region of the cargo Snurportin. We report two additional interaction sites between C-Tpr and Snurportin, suggesting a possible role for Tpr import into the nucleus. Using bioinformatics tools we have conducted conservation analysis and functional residue prediction investigations to identify which parts of the obtained binding sites are inherently more important and should be highlighted. Also, a novel measure based on the ratio of available solvent accessible surface (RASAS) is proposed for monitoring the ligand/receptor binding process.

## Introduction

The nucleus is the pivotal defining feature of eukaryotes, compartmentalizing the flow of information from DNA to protein by requiring that mRNA be exported to the cytoplasm prior to translation into proteins. RNA is exported across nuclear pore complexes (NPCs), mega-Dalton multi-protein assemblies embedded in the nuclear envelope, bridging the nucleoplasm and cytoplasm [1]. One of the major RNA-export pathways is the RanGTP-dependent pathway mediated by the exportin protein CRM1, also known as Exportin 1 or XPO1 [2].

During nuclear export, CRM1 first associates with RanGTP and the cargo NES (nuclear export signal) domain near the nuclear side of the pore complex. This complex migrates to the cytoplasm, where it encounters RanGTPases, whose dephosphorylation of RanGTP triggers immediate complex disassembly and cargo release. CRM1 then migrates back to the nucleus [3]. The scenario is similar to that for importins, which differs only in that the cargo is carried into the nucleus and that importin-cargo binding is independent of RanGTP [3], [4], [5], [6].

CRM1 exports a subset of mRNA, snRNP’s, some rRNA, and more than 200 proteins through their leucine-rich NES domain [7], [8], [9], [10]. It also has a critical role in the export of the RNA genomes of lentiviruses such as HIV [9], [11], . Moreover, many tumor suppressor proteins and cellular oncoproteins are dependent on CRM1 for their export [10], [13]. Thus, CRM1 could be an important target for therapeutic reasons, and many efforts have therefore been devoted to studying and developing inhibitory drugs that interfere with CRM1 binding [7], [10], [14], [15], [16].

Of particular interest in CRM1-dependent export is the protein Tpr, associated with the inner surface of the nuclear basket. Tpr, Translocated Promoter Region, is 2363 residues long, with a ∼1600 residue N-terminal domain composed of two parallel coiled coils and a highly acidic unstructured C-terminal domain [17], [18]. The C-terminus of Tpr comprises parts of the filaments extending from the nuclear basket, a structure on the nuclear side of the pore complex, while the N-terminus integrates into the basket itself [19]. The abrogation of proper Tpr expression by removal of its nuclear localization signal (NLS), preventing its localization to the nucleus, is known to cause a buildup of mRNA and leucine-rich NES-dependent proteins in the nucleus, suggesting that Tpr has a role in their export [20]. The particular classes of molecules affected by Tpr removal make it a prime candidate for interaction with CRM1, while its location suggests importance in the initial binding of CRM1 to the NPC.

Indeed, the potential role of Tpr in nucleocytoplasmic transport and its possible interaction with CRM1 has been debated in literature for over a decade, from both an in-vitro and in-vivo perspective [21], [22], [23], [24], [25]. While Tpr protein was identified for the first time more than 25 years ago [26], it was only in the late 90s that a role for Tpr in exporting mRNA was hypothesized [27], [28]. More specifically, in 2002 Shibata et al. found that poly(A) + RNA accumulated dramatically in Tpr-deficient nuclei, indicating a critical role for Tpr in RNA export regulation [21]. An independent study conducted by the Gerace lab in the same year substantiated the role of Tpr in nuclear export [23]. The authors found that the depletion of Tpr from the nucleus, while leaving the overall structure of the NPC and import of NLS-bearing cargos intact, markedly reduced the export of cargos containing leucine-rich NES. Among the best-known nuclear transport receptors of leucine-rich NES cargos is CRM1, and therefore, the possibility of an interaction between Tpr and CRM1 was suggested [23]. Seven years later, using solid phase binding assays, Ben-Efraim et al. were able to substantiate such an interaction with CRM1, in addition showing that Tpr binds to importin α and β[22]. They raised the possibility that Tpr provides a docking site for these transport receptors both in nuclear import and export.

On the other hand, Coyle et al. found that the CRM1-dependet export pathway is not sensitive to Tpr perturbation, suggesting at most a minimal role for Tpr in CRM1 export pathway [24]. This was further confirmed in a more recent study [25]. It appears that the time is ripe for a comprehensive atomic-level computational approach to investigating this challenging problem. The femtosecond-angstrom resolution of MD, absent in experimental studies, can serve as a powerful tool to investigate the possibility of such an interaction. Therefore, in this study we have used all-atom molecular dynamics to cast light on the details of the potential binding between CRM1 and human Tpr. We found that segmented peptides taken from C-Tpr show transitory binding to specific regions of the CRM1-RanGTP- Snurportin complex. Our results identify nine candidate binding sites on CRM1, as well as two additional candidates on Snurportin that predominantly bind to C-Tpr segments through salt bridges. To the best of our knowledge, this work is the first all-atom computational study targeting the specific interaction between Tpr and CRM1, which, while not providing a definitive proof of such an interaction, supplies a list of potential binding sites that will be useful for future studies.

## Methods

In this study we use molecular dynamics models to examine the details of CRM1-Tpr binding. After two sets of 200 ns long molecular dynamics simulations, interaction sites were selected using a combination of multiple criteria: visual inspection of how the export complex accommodates the C-Tpr fragments, the non-bonded interaction energy criterion, the energy landscape of interacting regions, change of surface accessibility and sequence conservation.

The large size of Tpr precluded a search for interactions over the entire surface of CRM1 using the whole Tpr, which would have necessitated repeated simulations with many copies of Tpr. Instead, the Tpr C-terminus was divided into 33 overlapping fragments. These peptides were then all placed randomly in proximity to the surface of CRM1-RanGTP-Snurpotin complex. This was done so that as many interactions as possible between C-Tpr and CRM1 were studied.

### Simulation Details

Molecular dynamics models were built using NAMD 2.7b2 [29] and the CHARMM27 force field [30], [31] to investigate the interaction between C-Tpr peptides and the CRM1-RanGTP-Snurpotin export complex. Unless otherwise indicated, protein manipulations, measurements, and water box addition were done with VMD1.8.7 and the included plugins [32].

Two independent simulations, each 200 ns long, as well as preliminary minimization and equilibration were performed. For our simulations, we use crystal structures for CRM1 with and without RanGTP (PDB accession: 3GJX, 3GB8) [2], [33]. Both structures contain Snurportin as cargo, which was left in since any CRM1-Tpr interaction should be studied in the presence of a cargo. Since the structure given is a dimer, only half of the PDB structure was used in simulation.

The unstructured carboxylic tail of Tpr, C-Tpr, residues 1700–2363, was built as an extended protein chain from amino acid sequence using the Pepbuild server [34]. This was then minimized for 500 steps in vacuum and 1 ns of equilibration to compact the structure into a system small enough to be simulated in reasonable time with explicit solvent. An explicit water box with a 5Å margin was then placed around it and another 1 ns of equilibration performed.

In order for the unstructured C-Tpr to have sufficient mobility and able to visit the entire exposed surface of CRM1 during the simulation time, we chopped C-Tpr into fragments (see Table S1). This approach enables residues of C-Tpr to have a higher chance to assume contact with CRM1 surface in a reasonable simulation time. This approach is, of course, applicable only to unstructured proteins, which inherently allow their residues to wander around the binding target. While we cannot consider this replacement as an ideal equivalent for the real system, previous works have proved it to be reliable and efficient [35], [36], [37]. The equilibrated C-Tpr chain was segmented into 33 fragments, each 30 to 44 residues in length, with extensions beyond 30 used to prevent the occurrence of structurally-important proline residues near fragment ends (see Table S1 for more details). These fragments had a deliberate overlap of 10 residues at both ends, to preclude the possibility of dividing a binding region in half.

**Figure 1:**
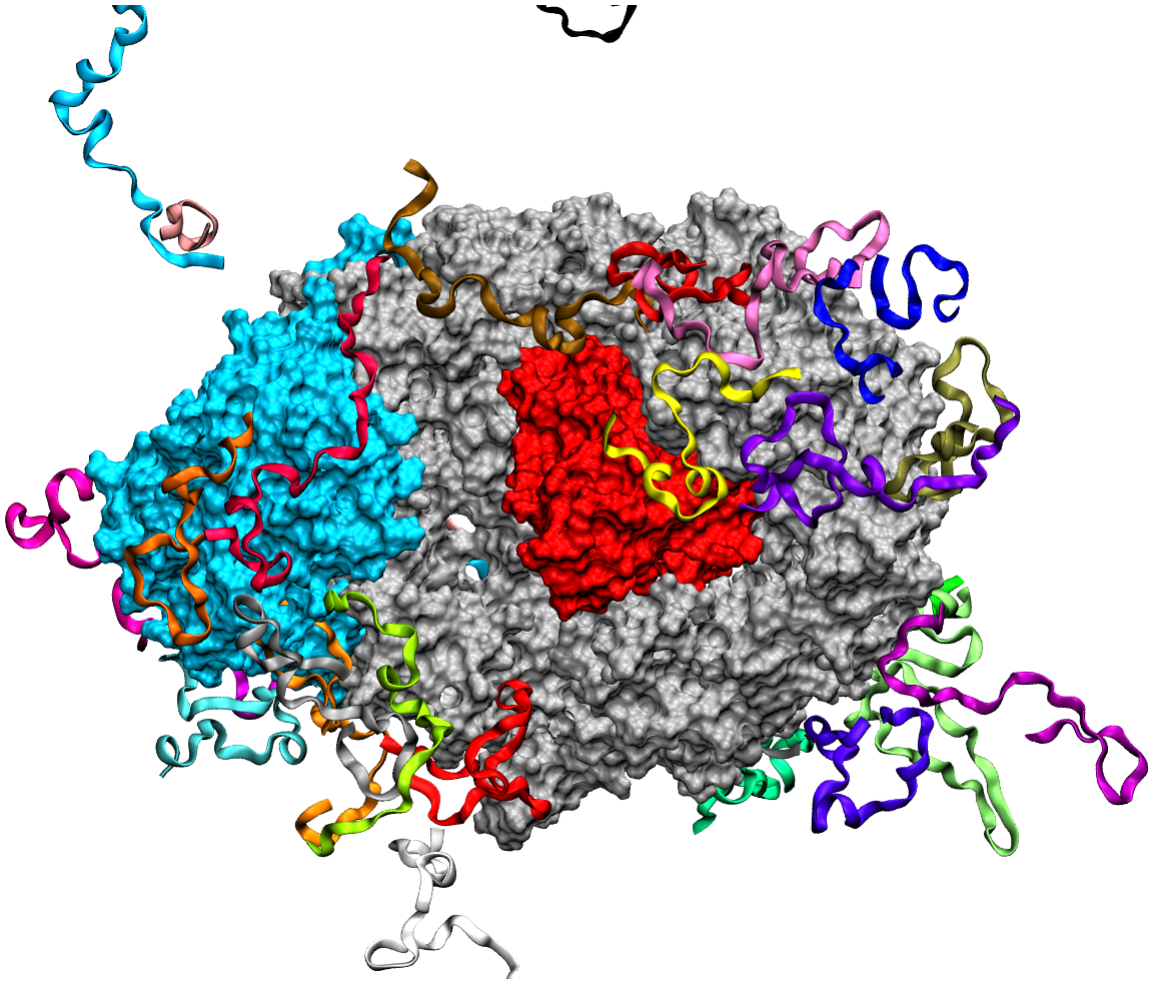
Simulation setup for binding of CRM1 and C-Tpr. Simulation Arrangement of C-Tpr fragments (ribbons) around the CRM1 (silver)-RanGTP (red)-Snurportin (cyan) complex after 200 ns.

Simulation systems were then assembled by arranging the 33 fragments of C-Tpr randomly around the surface of the CRM1-RanGTP-Snurportin complex (PDB accession: 3GJX) (See Figure 1 for a sample simulation result). This was performed manually using Swiss Viewer [38]. It must be noted that the fragments were arranged differently in two simulations in order to enhance the chance of Tpr encounter with the complex and cover larger areas of its surface. This system was then placed in a water box with the TIP3P water molecule, a 5 Å margin and Na^+^ and Cl^−^ counter-ions at the concentration of 100 mM.Periodic boundary conditions were used with the cell dimensions of 160Å×163Å×160Å and 145Å×143Å×182Å for the first and the second simulations respectively. In all simulations, Particle Mesh Ewald [39] was used for electrostatic energy calculation. Total atom numbers were 402,543 (simulation #1) and 362469 (simulation #2), within which the CRM1 complex and C-Tpr possess 24,083 and 15,704 atoms, respectively.

The simulation was initialized with a pre-equilibration procedure based on that of Isgro et al.[35]. Initially, everything but the water molecules were fixed, and the system minimized for 5000 steps and equilibrated for 4 ns. Then the CRM1 was fixed and this process repeated. Then the Tpr was fixed and the process repeated. Finally, this process was done on the entire system.

In order to conserve computation resources the bonds between hydrogens and larger atoms were held at fixed length, and thus, a timestep of 2 fs was used. The default multiple timestepping method of NAMD was used [40], with 2 fs step for bonded force evaluation, 4 fs for nonbonded forces, and 8 fs for long-range electrostatics. Pressure was regulated using a Langevin piston [41] period of 100 fs and damping timescale 50 fs, and a Langevin damping factor of 2 fs^−1^. Simulations were done in NPT ensemble. The cutoff for vdW was 12 A with switching distance of 10A. Langevin thermostat was used for controlling the temperature. Simulations were run for 200 ns at 310 K.

### Conservation Analysis

The binding events and corresponding binding sites were determined mainly based on the distance between interacting amino acids and energy of interaction. Moreover, by visual inspection we were able to unify some nearby interacting spots into a specific binding site. As an additional criterion for choosing binding sites, multiple sequence alignments (MSA) were conducted to specify the level of sequence identity at different binding spots. Protein sequences were obtained from UniProt Knowledgebase [42]. The selected homologs for sequence alignment are those with the exact name of the proteins of interest (CRM1 and Snurportin) and which are annotated manually (based on Swiss-Prot database [43]). Using this approach, we can confirm conservation of the relevant sequence between different species, which increases the likelihood of the binding site being significant. In addition, we can deduce conserved residues. Multiple sequence alignments were performed in JalVeiw [44] and based on ClustalW [45]. For CRM1 we aligned *M. musculus*, *R. norvegicus*, *H. sapiens*, *D. melanogaster*, *S. cerevisiae* and *S. pombe*. For Snurportin we considered *H. sapiens*, *M. musculus*, *R. norvegicus*, *B. taurus*, *G. gallus* and *D. discoideum*. Residues similar in charge and polarity are considered as conserved.

### Ratio of Available Solvent Accessible Surface (RASAS)

The average amplitude of interaction energy is a measure of binding strength. However, measurements of the interaction energy reveal little about geometry of interaction. Hence, we need a topological quantity to monitor how the ligand attaches to the receptor. Other details of the binding can be inferred from temporal behavior of the interaction energy.

Variation of the solvent accessible surface area (SASA) is a measure for the conformational change of proteins upon binding [46]. In addition to monitoring the conformational changes, SASA can be used to observe the binding events in a temporal fashion during the binding. We propose a measure based on SASA as a tool for tracking the binding. This quantity is the ratio of available solvent accessible surface (RASAS), which is defined as the ratio of SASA of a binding site in the presence of ligand to its amount in the absence of ligand. In other words, RASAS reports the portion of the binding site on the receptor which is not occupied by the ligand, and is intended to exclude the change in SASA due to the conformational changes. Essentially, with a probe radius of 1.4 Å, two SASAs are calculated for the binding site (or any selected region on the molecule) during the binding simulation: one is the actual SASA in the simulation, and the other is the SASA while ignoring the presence of ligand. RASAS is defined as the ratio of these two values.
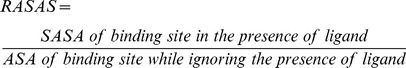
(1)Both numerator and denominator re recalculated for each MD snapshot. A lower magnitude of RASAS would imply higher binding site occupancy by the ligand. A value of 1 indicates no binding. While the energy drop shows the strength of the binding, RASAS assess the binding site coverage by the ligand from a topological viewpoint.

### Prediction of Functional Interfacial Residues

Various approaches exist for the prediction of functional and interfacial residues based on amino acid sequence and/or protein structures [47], which may be used as complementary tools to our simulations. Several web servers are available for this purpose (cons-PPISP [48], PIER [49], ConSurf [50], ProMate [51], PRISM [52], SPPIDER [53] and WHISCY [54]). We selected SPPIDER based on its superior performance in comparison to other servers [55]. In this work, we used SPPIDER I [53] with the tradeoff parameter of 0.3 to predict the residues in the complex which tend to be interfacial while interacting with other proteins. This tool works based on the prediction of solvent accessibility variation and uses neural-network method as learning tool.

## Results

Despite the pivotal role of CRM1 in nucleocytoplasmic export, the molecular details of the binding mechanism this exportin protein employs during export has remained unknown. In this study, we have developed molecular dynamics models to explore the details of CRM1-Tpr binding. Nine binding sites were identified on CRM1 as well as two additional sites on Snurportin that predominantly bind to C-Tpr. This study represents the first reported specific binding sites for Tpr on CRM1. Additionally, two regions of the exposed surface of Snurportin amenable to C-Tpr binding are identified.

### Summary of the Observed Binding Sites

All binding events observed in these simulations were pooled together. The obtained molecular dynamics trajectories were analyzed to identify possible bindings over the course of the simulation. Regions primarily selected to study were determined based on the distance criterion: individual fragments of C-Tpr were paired with residues of CRM1 that were within 7Å of the Tpr fragments. The 7 Å distance was chosen as a conservative threshold to determine the initial set of potentially interacting residues. For each fragment of C-Tpr, interaction energy was measured for all residues of CRM1 with a centroid within 7 angstroms any residue of the fragment (this selection was done with VMD). All regions with a drop in total nonbinging energy of 3 kcal/mol per residue or more were kept for analysis.

Then the Tpr fragments were went through manually, and broken up if it was clear that the interaction taking place was actually two (or more) interactions taking place in different parts of the Tpr fragment (i.e., there’s a set of residues in between the interacting residues that aren’t attached to Crm1). Then, the Crm1 residues involved in all the these interactions were examined, and if two groups of residues were obviously part of the same area (i.e. right next to each other). Afterwards, they, and their corresponding C-Tpr residues (which may be from more than one fragment) were merged into the same area. At each step, energy and RMSD was re-measured to make sure the interactions were still strong.

Visual observation, such as observation of apparent salt bridge formation and charged interactions, and measurements of physical proximity, were also used to help further inform the forming of sites. In other words, the merging or splitting of different candidate groups is carried out based on observations of highly active regions, and on observations of fragment arrangement on the surface. Upon definition of new sites, new interaction pairs were formed and interaction energies were recalculated. Regions with large and stable energy drops were included in the list of candidate binding sites, in some cases following additional visualization and adjustment of residues based on interaction energy. This led to the exclusion of the residues having very low energy contributions or located in positions that are not accessible. While low-interaction energy residues may still contribute to binding, we wished to focus on only the most important interfacial residues to ensure the validity of our list. Finally, the spatially adjacent sites were merged to gain new sites and the interaction energies were calculated based on the new set of residues in each site.

**Figure 2:**
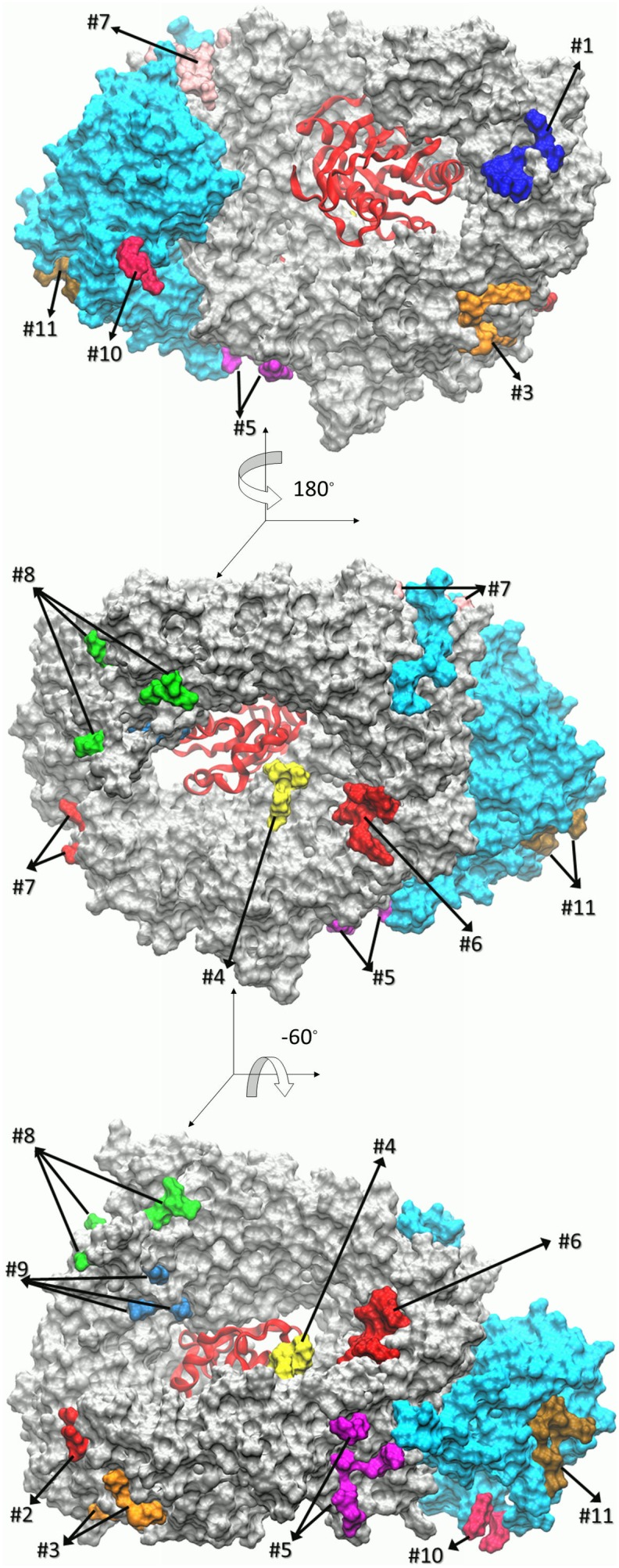
Location of C-Tpr binding sites on CRM1 (silver) and Snurportin (cyan) based on the collective results of two 200 ns-long simulations. (A) Solvent accessible representation of CRM1-RanGTP-Snurportin complex. Binding sites are numbered according to their position in the amino acid sequence (see Table 1) with different colors. RanGTP is shown as a red ribbon. (B) Angle of view is rotated 180° about the vertical axis. (C) Angle of view rotated −60° about the horizontal axis.

**Figure 3:**
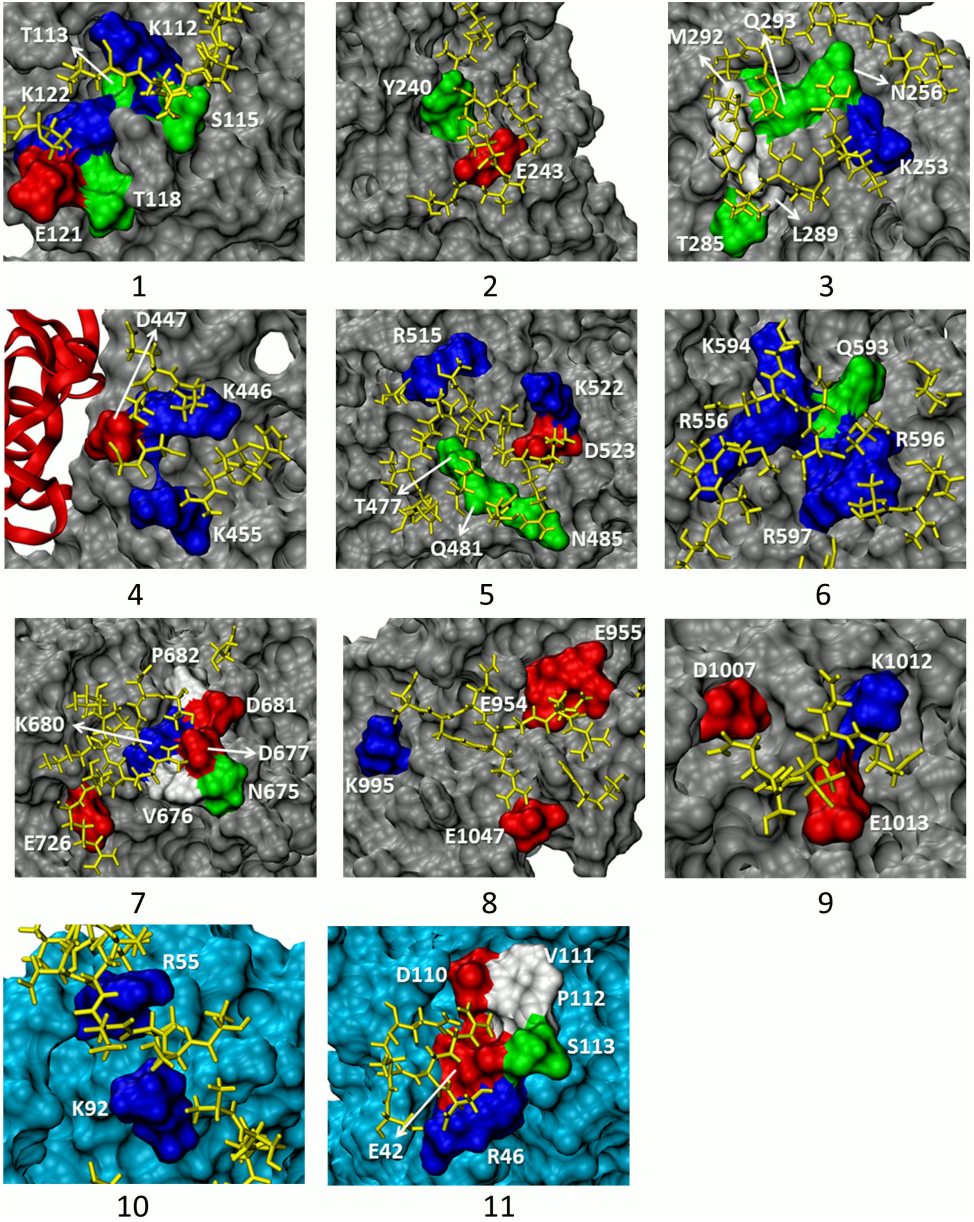
Close-up views of nine binding sites on CRM1 (1 to 9) and two sites on Snurportin (10, 11). The most important residues involved in binding event are colored based on their charge attribute: red for acidic, blue for basic, green for polar, and white for nonpolar. Other parts of CRM1 are colored in silver and the C-Tpr fragments are in yellow. Note that only the closest C-Tpr residues are depicted. It can be seen that binding sites are mainly composed of charged and polar residues. The first three sites are located far away from the Snurportin and on the convex side. Site 4 is next to the RanGTP-CRM1 concave side. Sites 5 and 6 are both located close to the Snurportin. The former lies adjacent to the Snurportin-NES binding site on the convex side and the latter rests on the concave side of the CRM1. Site 7 is located very close to the Snurportin on the CRM1 convex side while site 8 is far from it. Site 9 is next to the RanGTP on the CRM1 concave side away from the Snurportin. This site is blocked by the helix H20B in the absence of the RanGTP. Sites 10 and 11 are on the Snurportin. The coloring scheme of the binding residues is the same as those on the CRM1. The rest of the Snurportin is colored cyan. Site 10 lies close to the convex side of the CRM1, while site 11 is far from the CRM1.

Table 1 summarizes the nine binding sites on CRM1 and two on Snurportin as well as their characteristics, including average values for interaction energy, RASAS, and locations on the complex. Because of the obvious differences between the two simulations, they are not expected to give identical results. They are, however, considered complementary. The size of the binding regions varied from two up to seven residues. Figure 2 depicts the location of the proposed binding spots which spread all around the complex. Figure 3 gives a closer view of the binding sites and adjacent C-Tpr residues. Some are connected (e.g. 1, 3 and 6) while others are disconnected (e.g. 5, 8 and 9). Although some may look quite dispersed (e.g. site 8), they are counted as a single site, based on the observation of simultaneous binding to a single Tpr fragment, suggesting possible complimentary binding. In addition, the corresponding average energy drop of site 8 shows strong binding in both simulations, and all of the listed residues contained substantial interaction energy. In our binding of interest, there is no apparent structural matching and ligand/binding pocket fitting that we usually see in complex formations. Instead, we have dispersed attachments and detachments of C-Tpr to the export complex. In addition, the unstructured nature of C-Tpr prevents its segments from binding in an organized and ordered manner.

**Table 1:** A summary of 11 C-Tpr binding sites on CRM1-RanGTP-Snurportin complex predicted based on our simulations.

Site #	Residue of CRM1/Snurportin*	Energy (kcal/mol) (Simulation #1)	Energy (kcal/mol) (Simulation #2)	RASAS (Simulation #1)	RASAS (Simulation #2)	Position
1	K112† T113 **S115 T118 E121** † **K122**	−51.91±44.01	−151.79±51.69	0.79±0.09	0.51±0.09	Far side away from the Snurportin, on convex side
2	Y240 E243 †	−131.36±23.60	−83.90±60.09	0.46±0.13	0.46±0.13	Far side away from the Snurportin, on convex side
3	**K253** † **N256** T285 L289 M292 Q293	−69.37±32.53	−18.90±7.86	0.85±0.08	0.58±0.09	Far side away from the Snurportin, on convex side
4	**K446** † **D447** K455†	−70.70±72.48	−8.19±20.41	0.83±0.18	0.99±0.03	Next to the RanGTP, concave side
5	**T477 Q481** N485 **R515 K522 D523**	−20.77±7.51	−24.79±42.40	0.67±0.06	0.73±0.11	Next to the Snurportin-NES binding site, on convex side
6	R556 † Q593 K594 R596 † R597†	−223.59±107.39	−223.17±63.76	0.64±0.15	0.66±0.08	Near Snurportin, on the concave side
7	N675 **V676** D677† K680 D681P682 E726†	−79.66±41.98	−0.27±3.46	0.70±0.10	1.00±0.01	Immediately next to Snurportin on convex side
8	**E954** † **E955** † **K995 E1047** †	−125.01±44.76	−127.33±67.04	0.78±0.09	0.87±0.07	Far side away from Snurportin
9	D1007† K1012 E1013	−58.68±42.29	−60.00±44.12	0.80±0.12	0.92±0.06	Next to RanGTP, concave side, away from Snurportin; blocked by helix H20B in absence of RanGTP
10	**R55** * † K92 * †	−180.08±70.77	−37.86±31.57	0.44±0.14	0.72±0.14	Side of Snurportin near convex side
11	**E42** * † **R46** * D110 * † V111 * P112 * S113*	−155.62±51.86	−0.54±5.73	0.69±0.06	0.98±0.03	Far edge of Snurportin

Binding sites are sorted according to their position in the amino acid sequence. There are nine binding sites on CRM1 with the total number of 42 residues and two binding sites on Snurportin with the total number of eight residues. In the second column, functional residues predicted by SPPIDER I, salt bridge-making residues, conserved residues, and residues belonging to Snurportin are distinguished by different notations (see below). Moreover, the average interaction energy and RASAS are shown for two simulations in next columns. Notations:

**Bold face**: Residues which were predicted by SPPIDER as functional interfacial amino acids (21 AAs).

† Residues which form a salt bridge (19 AAs). The cutoff is set to 3.2 Å.

Underlined: Residues which are fully conserved (20 AAs).

*Residues from Snurportin.

**Figure 4:**
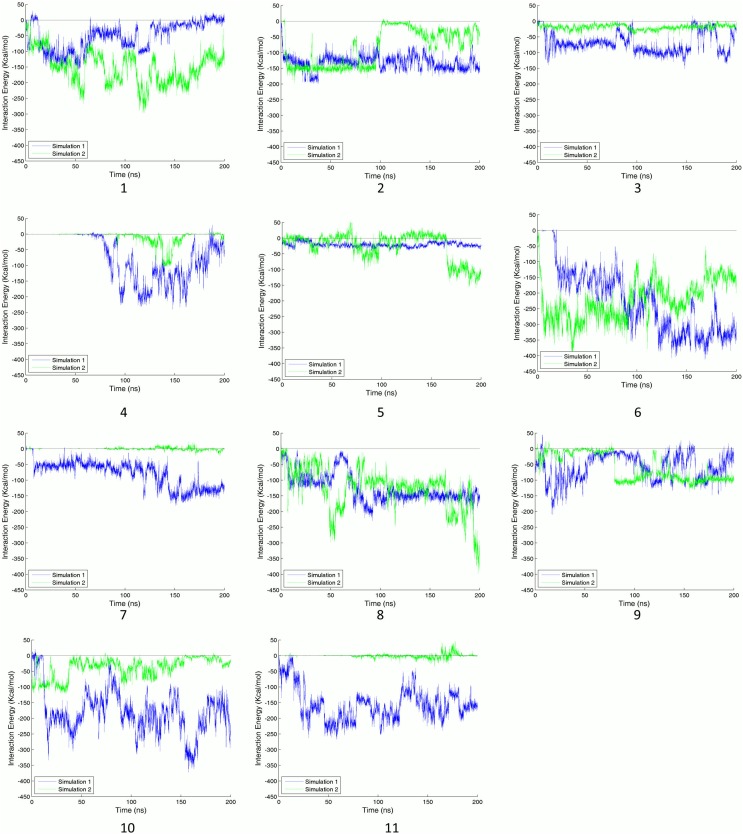
Nonbonded interaction energy between binding sites and C-Tpr fragments. The number of the binding site appears under each graph. Graphs for simulations 1 and 2 are plotted in blue and green lines, respectively. A significant stable energy drop is the main factor for detecting a reliable binding site. Although some of the reported binding sites satisfied this criterion for both simulations, not all of them showed significant drops in both simulations, which is quite normal due to the finite time of the simulations.

The reported sites possess a wide range of average energy and RASAS. However, the average values cannot represent the transient details of a binding process. Interaction energies between binding sites’ residues and their adjacent C-Tpr fragments are shown in Figure 4. An apparent energy drop is not necessarily present in both simulations. While sites 1, 2, 3, 6, 7, 8, 10 and 11 show stable and strong interactions with C-Tpr fragments, others lack either perfect stability or strength. Nonetheless, for the sake of having an inclusive list, sites 4, 5 and 9, which have only partial stability, are also considered as candidate sites. Binding sites 4 and 5 are among the most prominent, when considering sequence conservation and the presence of predicted hot and functional spots by SPPIDER. Site 4 is also host for two salt bridges, while site 5 shows a stable drop in RASAS, which is evidence for the presence of Tpr in the binding site throughout the simulation (refer to the following sections for details).

### Sites Close to Snurportin

There are three sites flanking Snurportin binding region: The strongest site (site 6), a site with a moderate strength (site 7) and a site with a relatively weak interaction (site 5). Interestingly, these sites are also located in the vicinity of the binding site for any standard NES-bearing cargo – which differs from the main interfacial region between Snurportin and CRM1 [33] – and could be plausibly affected by binding there. This result suggests that the presence of some form of cargo is important to CRM1/C-Tpr binding. Notably, the nearby regions of Snurportin itself do not appear to play a significant role in the binding interactions. Given that the majority of CRM1 cargos rely on a short NES-recognition motif to bind to CRM1, it seems unlikely that the remaining portions of cargo, which vary from cargo to cargo, participate directly in in CRM1-Tpr binding. It is, however, possible or even likely that cargo binding triggers reinforcing allosteric effects, via shape changes in CRM1.

### Sites Adjacent to RanGTP

Sites 4 and 9 are close to RanGTP and, indeed, some residues of the RanGTP are involved in binding (e.g. residue K99). Nonetheless, these are not listed as binding site constituents because of their lower contribution relative to CRM1 residues. However, the presence of RanGTP and the resulting conformational changes conceivably have a role in CRM1/C-Tpr interaction. These binding sites would not likely materialize in the absence of RanGTP. This implies that CRM1, like other exportins involved in the RanGTP-dependent cycle, can exit the nucleus only when bound to RanGTP [4]. Thus, sites 4 and 9 likely help to mediate CRM1/C-Tpr interaction only in the presence of RanGTP.

Besides relying on residue K99 of RanGTP, interaction with the residues of site 9 appears to be blocked in the absence of RanGTP. Specifically, in an equilibrated crystal structure without RanGTP, but containing Snurportin, residue K1012 of site 9 has a charged interaction with residue E1036 of CRM1 in HEAT helix 20B. This interaction appears to block entrance of other interaction partners. Conversely, in an equilibrated structure containing RanGTP, this same helix is moved dramatically out of the way, rotating almost a full turn, which uncovers the binding site (see Figure S1).

### Sites on Snurportin

Two sites (10 and 11) were also identified on Snurportin, an unanticipated result, given that this was not our expected interaction (Figures 2 and 3). Given its role here as a cargo [2], [33], we conclude that Snurportin cannot specifically be more important to export than any general type of cargo. Snurportin, however, is itself an importin, and we speculate that this interaction might have a role in Snurportin-dependent import, or be the last stage of Snurportin-NPC interaction. It must be noted that the sites found here, show noticeable interaction energies compared to most of the observed sites on CRM1.

### RASAS as a Promising Binding Signature

**Figure 5:**
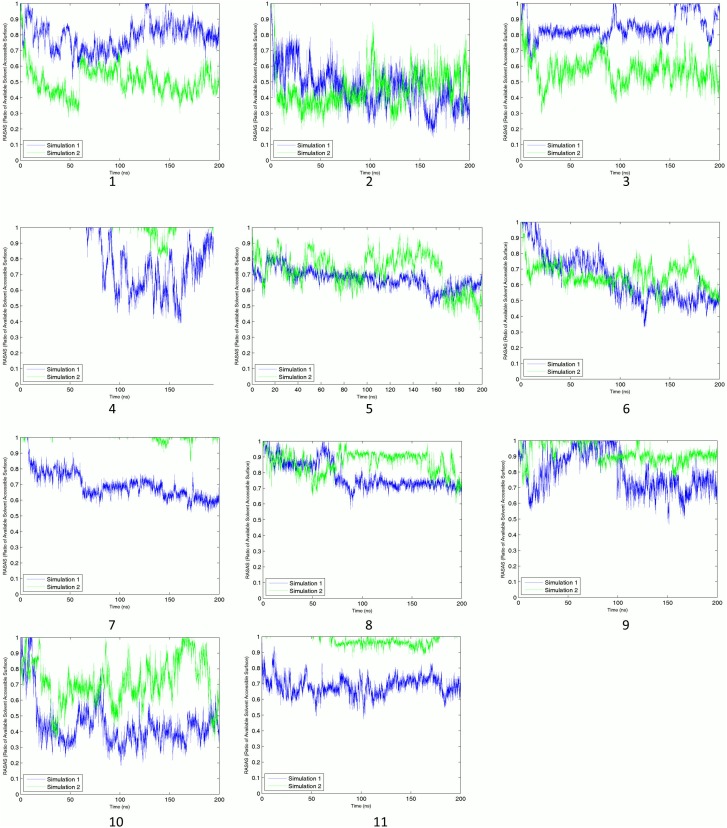
Ratios of available solvent accessible area (RASAS) for binding sites. Binding site number appears under each graph. Graphs for simulations 1 and 2 are plotted in blue and green lines, respectively. Although the nature of RASAS is completely different from the interaction energy, they are generally in agreement, building confidence in the results.

In addition to having a notable energy drop (see Figure 1), the observation of RASAS drop – which is interpreted as interfacial contact – was also considered as a characteristic of a binding event (see Figure 5). As a complementary criterion, RASAS ratios (see Equation 1) were calculated for the identified binding sites based on side-chain SASAs. Average values of this quantity are reported in Table 1 for different sites for two simulations. Although only the trend of RASAS over time can tell us the whole binding story, the mean values allow for a quick comparison among various cases. Sites 1, 2, 3 and 10 have the lowest RASAS either for one or two simulations. While there is a good qualitative match between RASAS and the interaction energy for each binding site, there is not necessarily a direct correlation between their mean values. For example, while site 6 has the highest interaction energy, its mean RASAS value is not the lowest.

Similar to energy profiles, RASAS profiles are depicted for binding sites during the course of both simulations (see Figure 5). Even though RASAS and interaction energy are totally different in nature, our results show that they are in agreement with each other for binding sites 4, 6, 7, 8, 9, and 11 in both simulations, offering more confidence in these binding sites. For these cases, most of the energy upward and downward trends can be observed in their corresponding RASAS profiles. For other binding sites, however, there is only partial agreement between RASAS and energy drop, yet still notable. For examples, for sites 2, 3 and 10 we could only observe the RASAS-energy drop agreement in one of the simulations. This level of agreement suggests that RASAS can be considered as a promising measure for quantifying the degree of binding. Although RASAS was not the main criterion for judging the binding strength, it can tell us about the proximity of interacting partners. For instance, in spite of some ups and downs in the energy profile for site 6, RASAS adds to our confidence in the interaction by showing a stable drop, indicating a steady contact between the interacting agents.

RASAS can also give us a measure of site occupancy time. With a 1.4 Å probe radius, whenever the RASAS goes below 1, the ligand is within a 2.8 Å proximity of the binding site. By observing the RASAS plots we can see that in almost all cases, there is a Tpr fragment present in the neighborhood of the binding site. The only exceptions are site 4 in both simulations and sites 7 and 11 in the second simulation. The RASAS formalism was also used to monitor the portion of the complex surface covered by the peptide chains. Interested readers may refer to Figure S2 for further details.

### Binding Sites’ Conservation

Multiple-sequence alignment was used to determine the level of conservation for each binding site. This criterion was not a main factor for discrimination in favor of some binding sites, but provides some insight into the importance of binding regions. Details of alignment are shown in Figures S3 and S4 for CRM1 and Snurportin, respectively. The most apparent conserved portion of CRM1 is the binding site for cargo NES (i.e. from 500 to 580) [56]. In addition, there are some conserved regions on the concave side which serve as RanGTP binding domain. Most of the amino acids of Snurportin are conserved (sites 10 and 11). However, CRM1 shows a broader range of conservation profiles. Site 2 is fully conserved, while sites 3, 4, 5, 6 and 9 are mostly conserved. The remaining sites (1, 7 and 8) possess a moderate level of conservation among different homologs. From the total of 50 residues in binding sites, 21 of them are fully conserved. Evolutionary change in a residue to another residue from the same group (acidic, basic, polar uncharged and nonpolar) is considered as conservation.

The binding sites on CRM1 generally show the same conservation level as the other parts of this protein. However, it is apparent that the most critical segments, including RanGTP and the NES binding sites, are conserved better than the other regions. Still at least one fully conserved residue is present in 8 out of 9 sites on CRM1. The sites on Snurportin are as conserved as the rest, generally showing high levels of conservation.

### Prediction of Interfacial Residues

A list of predicted interfacial residues was obtained using SPPIDER I [53] for both the CRM1 and the Snurportin based on their complex crystal structures. As shown in Table 1, there is a common subset between the simulation outcome and predicted interfacial residues. Although less than half of the binding sites’ residues were predicted as interfacial ones for CRM1 (21 out of 50 AAs for the whole complex), most of our proposed binding sites contain at least one predicted interfacial residue (8 out of 11 total binding sites). Interestingly, despite their marginally stable interaction energy landscapes, sites 4 and 5 have the highest rank in the prediction, and thus we kept them in the list of potential binding sites.

### Structural Flexibility

**Figure 6:**
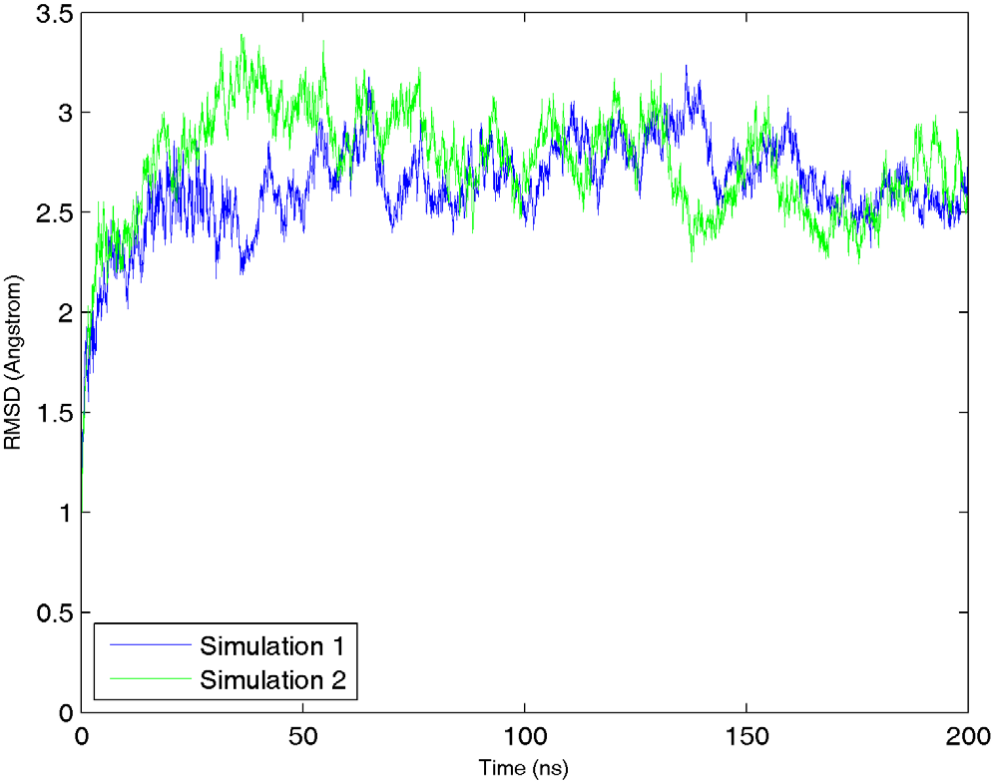
RMSD plot for the CRM1-RanGTP-Snurportin complex in two simulations. The minimized structure of the export complex is used as the reference. Because the system has already equilibrated, after a short time in simulation the RMSD reaches its stabilized value between 2.5 and 3 Å.

**Figure 7:**
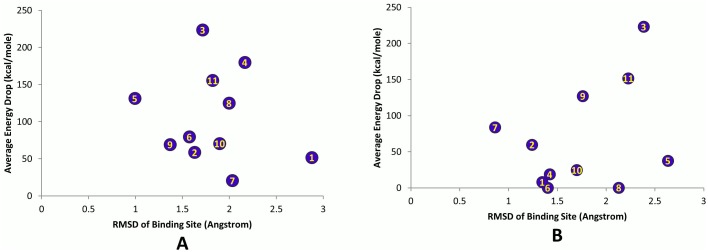
The average energy drop against RMSD of the binding sites. (A) Simulation #1. (B) Simulation #2.

**Figure 8:**
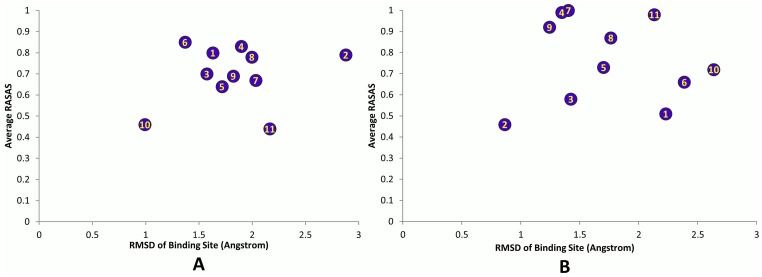
The average RASAS drop versus the RMSD of the binding sites. (A) Simulation #1. (B) Simulation #2.

The root mean square deviation (RMSD) of the whole export complex stabilized between 2.5 and 3 Å, showing acceptable stability over the course of simulations (see Figure 6). In addition, RMSD was calculated for each individual binding site. Its averaged values are plotted versus the average interaction energy and RASAS in Figures 7 and 8. We did not find any apparent correlation between the RMSD and the interaction energy or RASAS.

### No Clear Binding Motif

**Figure 9:**
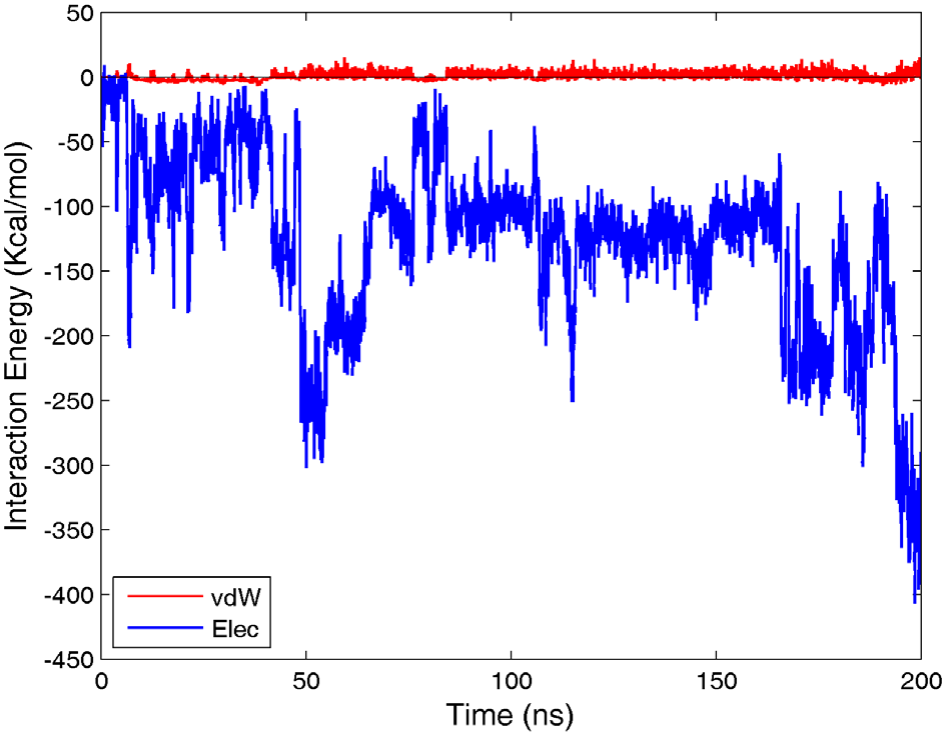
Nature of interaction energies. As a representative sample, the electrostatic and van der Waals interaction energies between site 8 and the C-Tpr fragments are shown (simulation #2). As it can be seen, the van der Waals energy is negligible compared with electrostatic Thus, the nature of interactions is mainly electrostatic.

Our investigations find no evidence of a clear motif within the sequence of C-Tpr. Rather, it can only be said that charged residues such as arginine, aspartate, and glutamate appear to play an important role, with the basic residues in particular being central to interaction with the outer loop regions of CRM1. As a result of containing various acidic and basic residues in the binding sites, salt bridge formation plays an important role in bindings. During the course of simulations, 19 out of 50 participating residues formed salt bridge with their corresponding partners on C-Tpr fragment (see Table 1). Expectedly, interaction energy profiles show a clear dominance of electrostatic over van der Waals energy (see Figure 9 for an illustrative example). C-Tpr contains three FG-motifs, and regions of CRM1 possess significant sequence homology with regions of Importin-β known to contain FG-repeat binding sites [35]. However, despite deliberate attempts to induce interaction between these repeats and the homologous regions on CRM1, we did not observe any binding. It appears that the FG-repeats in C-Tpr may not serve a critical role in relation to CRM1, unlike their function in FG-Nups. The presence of these three FG-motifs in C-Tpr, however, might have another implication about the potential role of Tpr in the import of Snurportin and other karyopherins (see Conclusions).

### Relevance to Other Types of Binding

Our study of CRM1-Tpr binding has revealed a small variety of candidate binding sites on the CRM1 surface. The existence of these interactions, and the strong effect of RanGTP on the binding sites found, lend further support for the hypothesis that Tpr acts as an early entry binding site for CRM1, as also implied by experimental evidence [17]. The interactions found likely furnish useful information about the properties of this kind of binding in general, especially since functional considerations suggest relatively promiscuous binding at the NPC periphery, due to the variety of different transport proteins that would need to bind [4], [57]. In particular, it is reasonable to speculate that the electrostatic nature of the CRM1binding sites also make them amenable to other unstructured regions in the NPC with similar charge properties to the regions on C-Tpr, but this was not tested in this study. Based on the obtained results, CRM1 seems to have a group of attractive sites for various regions of Tpr. However, on Tpr’s site, there is no specific site.

## Discussion

Our investigation binding dynamics of C-Tpr has furnished four primary results: i) a protein structural study of export complex to the periphery of the NPC, ii) a method of determining binding sites when one protein is unstructured and there is no well-defined binding motif, iii) a specific set of binding sites for C-Tpr and CRM1 in particular, and iv) a new measure called RASAS for monitoring the coverage of the ligand binding site on the receptor. The first of these, to the best of our knowledge, has not been previously done. In addition, our study suggests that Snurportin, itself an importin, binds to C-Tpr, a behavior that could potentially be shared by other importins.

Besides our quantitative analyses, several qualitative visual observations were made which, while not definitive, can serve as the basis for future studies. For example, widely disparate regions of C-Tpr can interact with CRM1, instead of the certain specific regions that might be expected. There exist, however, large, highly acidic regions of C-Tpr which resist interaction with CRM1, appearing to actively shift the fragments they are part of far away from the main body of CRM1. This suggests that not all of C-Tpr is functionalized for interacting with exportins, raising the possibility that the non-interacting sites serve some other, unknown purposes.

The side of CRM1 to which Snurportin binds–sites 5, 6 and 7–appears to be highly favorable to C-Tpr adhesion and aggregation (See Movie S1). On the concave side of CRM1, C-Tpr fragments adhere strongly to the inner surface (See Movie S2). Along with the location of various binding sites, these observations are consistent with the presence of cargo and RanGTP being important to binding, as reported experimentally [17].

Additionally, it is interesting that Snurportin, which was only included in the simulation system to fill the role of cargo, exhibited a significant binding partnership with C-Tpr. It cannot be expected that cargo interactions with C-Tpr would play any vital role in transport, but it is notable that Snurportin is itself an importin. Combined with the fact that Snurportin binds to the same overall regions of C-Tpr as CRM1 does, a possible role for C-Tpr during Snurportin-mediated nuclear import can be hypothesized. This notion is further substantiated by the fact that there are three FG-motifs in C-Tpr. Particularly, crystallographic studies suggest that karyopherins have up to several hydrophobic binding pockets on their outer surface to interact with FG-repeat domains [58]. Given the localization of C-Tpr to the distal ring, it can be speculated that interacting with C-Tpr is the last stage of the Snurportin-cargo complex journey to the nucleus. Indeed, that would be an interesting study to explore interactions of a number of karyopherins with C-Tpr chain to intensify this speculation.

Finally, it should be emphasized that the list of binding regions reported here may not be exclusive, since due to limitations of current methods it was not feasible to test all possible interactions of C-Tpr fragments with the CRM1 surface. We hope this partial list of binding sites may serve future studies. The only experimental evidence is the research that solely verifies the interaction between two proteins [17]. No specific evidence is available regarding the specific binding sites and modes. Our general suggestion is performing mutagenesis experiment (as a standard approach for such purpose) to examine the proposed binding sites.

## Supporting Information

Figure S1 **An overlapped image of the structure of CRM1 with and without RanGTP.** CRM1 is orange, RanGTP is red, and snurporitin is yellow. The structure containing RanGTP is transparent (though the blue regions are opaque for clarity), while the non-transparent structure lacks RanGTP. In the non-RanGTP structure, HEAT helix 20B, the cylinder colored in green, contains a residue E1036 (in green, with Van der Waal’s radius) which blocks residue K1012 of site 9 (also green). In the Ran-GTP structure, HEAT helix 20B, with residue E1036, and residue K1012 (both blue) are disassociated, with helix 20B shifting out of the way, and K1012 is exposed. Arrows show the movement of these areas when RanGTP is included.
(TIF)

Figure S2 **The percentage of surface coverage was calculated based on 1-RASAS.** The probe radius is set to 2.5 Å which is equivalent to a proximity distance of 5 Å. In this way the percentage of the complex surface covered by the Tpr fragments up to a 5 Å cutoff is calculated throughout the simulations. The total covered area rises to higher values and after the system gets stable is around about 30 to 35% in each simulation.
(TIF)

Figure S3 **Multiple sequence alignment for CRM1 and its functionally confirmed homologs.** Darker blue shows higher conservation rate based on the sequence identity. The column(s) above each red box shows the binding sites predicted by the MD simulation in the current study.
(TIF)

Figure S4 **Multiple sequence alignment for Snurportin and its functionally confirmed homologs.** Darker blue shows higher conservation rate based on the sequence identity. The column(s) above each red box shows the binding sites predicted by the MD simulation in the current study.
(TIF)

Table S1 **List of 33 C-Tpr fragments used in simulations.** To eliminate the possibility of dividing a binding region on C-Tpr, fragments have a 10 residues overlap at both ends, adjusted as necessary to avoid proline residues.
(DOCX)

Table S2 **List of the Tpr fragments interacting with each binding site.**
(DOCX)

Movie S1 **A view of the Snurportin side of CRM1 throughout simulation 1.** Snurportin is in yellow, CRM1 in orange, RanGTP in red, and C-Tpr fragments in grey. It can be seen that C-Tpr fragments approach and adhere to sites 5, 6, and 7 near Snurportin.
(MP4)

Movie S2 **A view of the concave side of CRM1 throughout simulation 1.** CRM1 is in orange, RanGTP in red, and C-Tpr fragments in grey. C-Tpr fragments can be seen adhering to the inner surface of CRM1.
(MP4)
