# Supplementary figures and images for: The Interaction of CRM1 and the Nuclear Pore Protein Tpr
Source: PLoS One. 2014 Apr 10;9(4):e93709. doi: 10.1371/journal.pone.0093709 (PMC3983112; doi:10.1371/journal.pone.0093709)

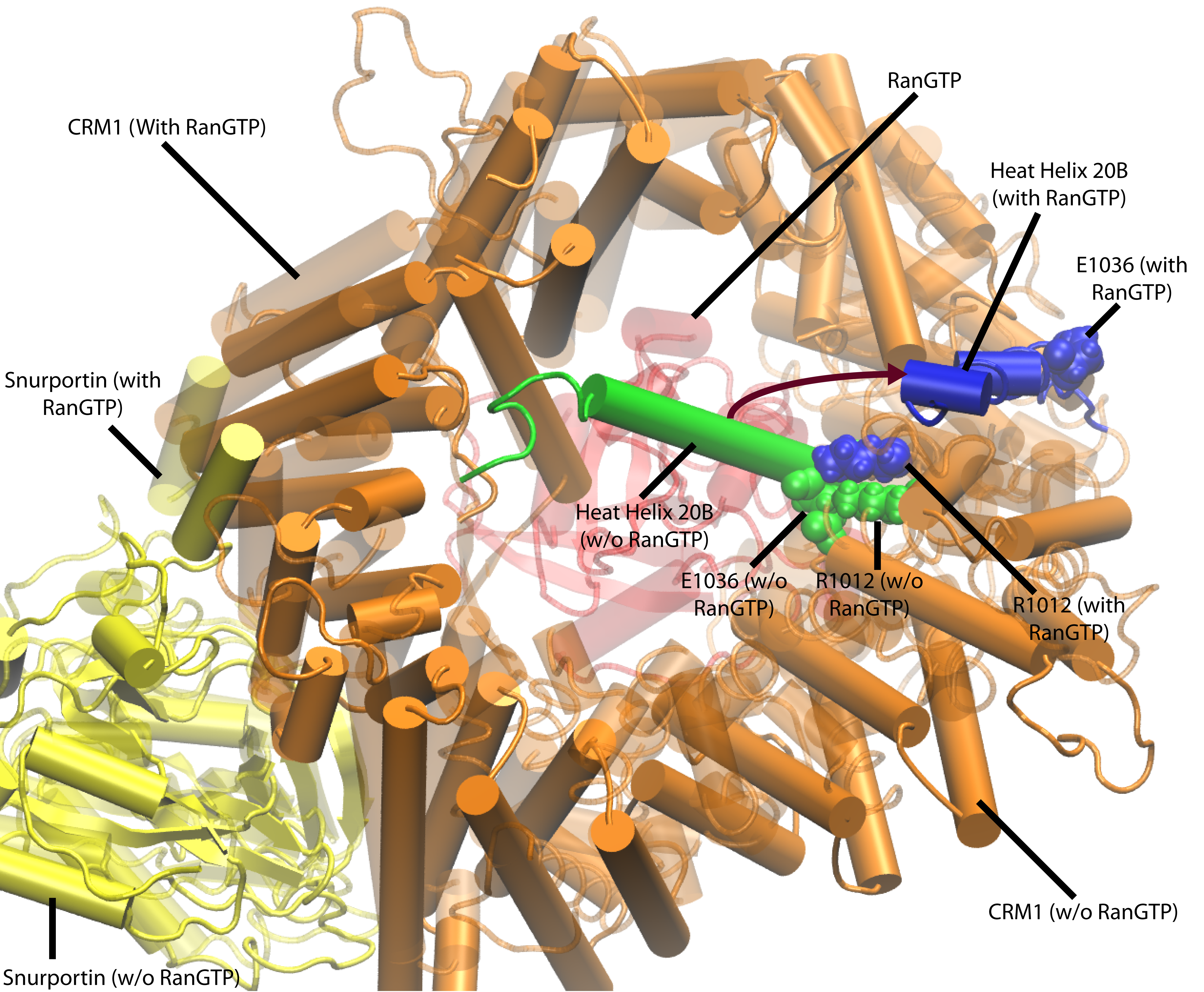

Supplement: Figure S1 — An overlapped image of the structure of CRM1 with and without RanGTP. CRM1 is orange, RanGTP is red, and snurporitin is yellow. The structure containing RanGTP is transparent (though the blue regions are opaque for clarity), while the non-transparent structure lacks RanGTP. In the non-RanGTP structure, HEAT helix 20B, the cylinder colored in green, contains a residue E1036 (in green, with Van der Waal’s radius) which blocks residue K1012 of site 9 (also green). In the Ran-GTP structure, HEAT helix 20B, with residue E1036, and residue K1012 (both blue) are disassociated, with helix 20B shifting out of the way, and K1012 is exposed. Arrows show the movement of these areas when RanGTP is included. (TIF) [file pone.0093709.s001.tif]

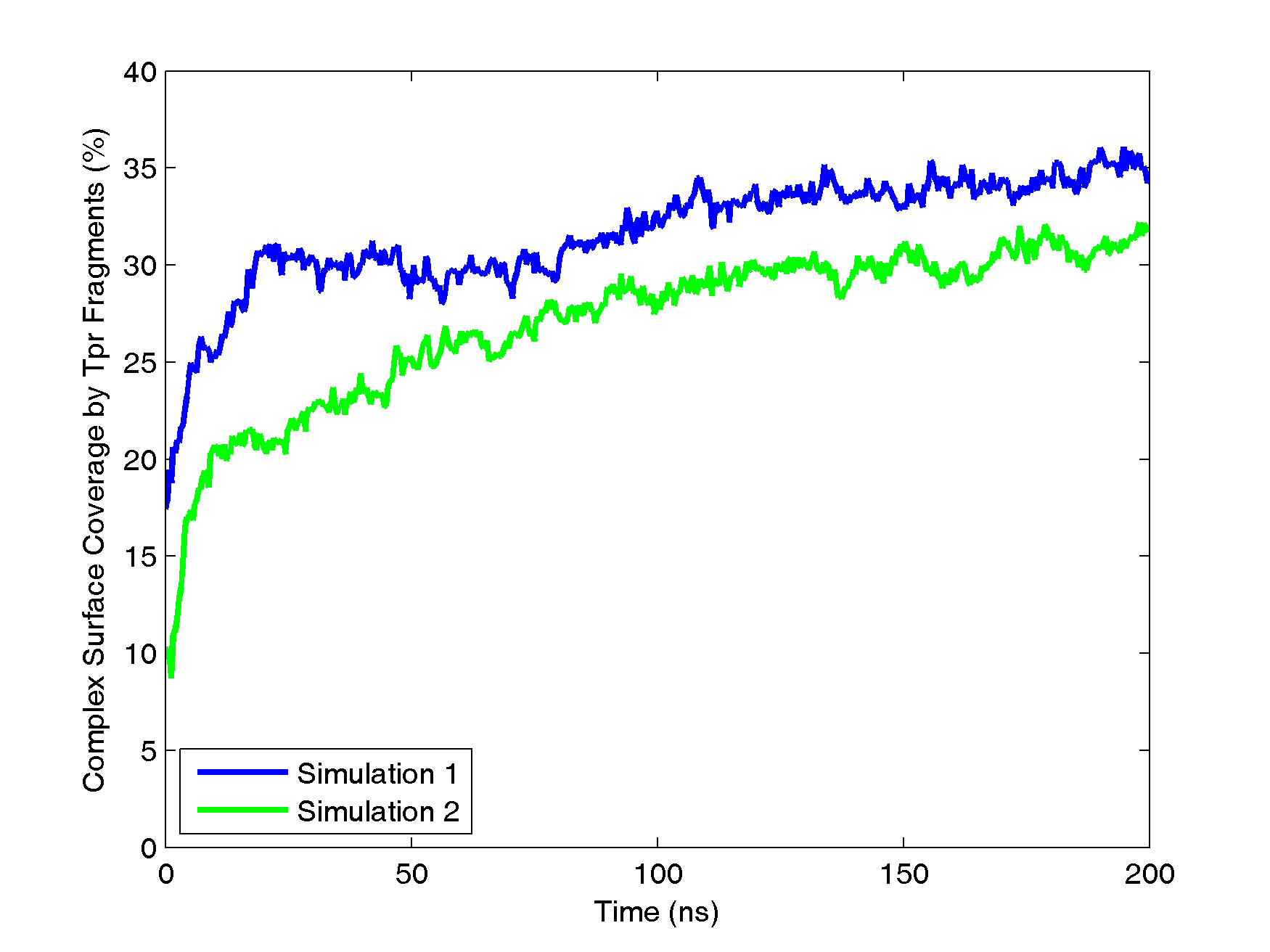

Supplement: Figure S2 — The percentage of surface coverage was calculated based on 1-RASAS. The probe radius is set to 2.5 Å which is equivalent to a proximity distance of 5 Å. In this way the percentage of the complex surface covered by the Tpr fragments up to a 5 Å cutoff is calculated throughout the simulations. The total covered area rises to higher values and after the system gets stable is around about 30 to 35% in each simulation. (TIF) [file pone.0093709.s002.tif]

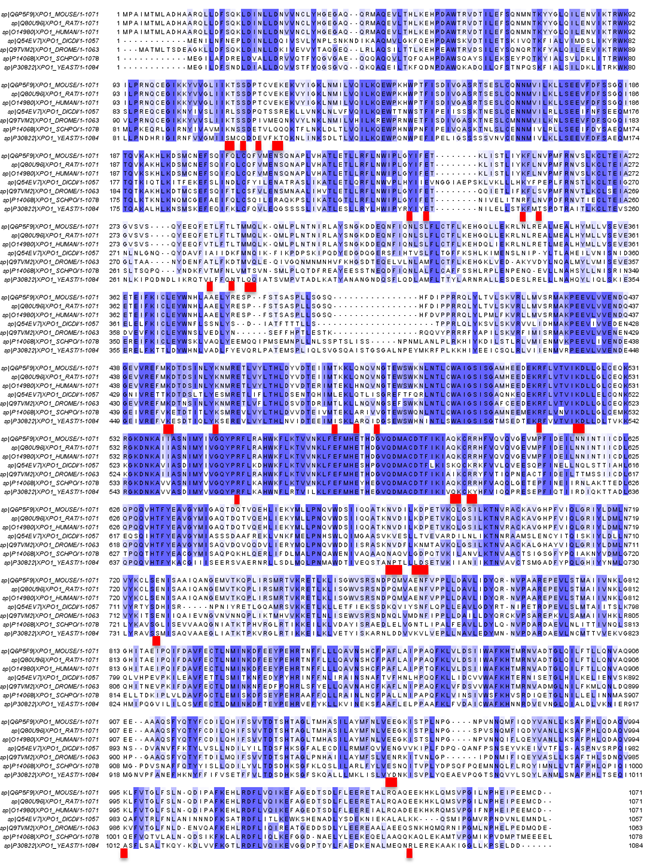

Supplement: Figure S3 — Multiple sequence alignment for CRM1 and its functionally confirmed homologs. Darker blue shows higher conservation rate based on the sequence identity. The column(s) above each red box shows the binding sites predicted by the MD simulation in the current study. (TIF) [file pone.0093709.s003.tif]

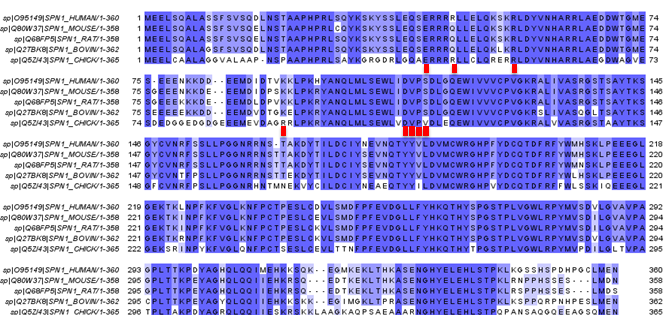

Supplement: Figure S4 — Multiple sequence alignment for Snurportin and its functionally confirmed homologs. Darker blue shows higher conservation rate based on the sequence identity. The column(s) above each red box shows the binding sites predicted by the MD simulation in the current study. (TIF) [file pone.0093709.s004.tif]
