# Supplementary material for: The Interaction of CRM1 and the Nuclear Pore Protein Tpr
Source: PLoS One. 2014 Apr 10;9(4):e93709. doi: 10.1371/journal.pone.0093709 (PMC3983112; doi:10.1371/journal.pone.0093709)
Supplement: Table S1 — List of 33 C-Tpr fragments used in simulations. To eliminate the possibility of dividing a binding region on C-Tpr, fragments have a 10 residues overlap at both ends, adjusted as necessary to avoid proline residues. (DOCX) [file pone.0093709.s005.docx]

**Supporting Information**

**Table S1:** List of 33 C-Tpr fragments used in simulations. To eliminate the possibility of dividing a binding region on C-Tpr, fragments have a 10 residues overlap at both ends, adjusted as necessary to avoid proline residues.

| **Segment index** | **1** | **2** | **3** | **4** | **5** | **6** | **7** | **8** | **9** | **10** | **11** |
| --- | --- | --- | --- | --- | --- | --- | --- | --- | --- | --- | --- |
| **No. of AA** | 30 | 30 | 30 | 46 | 36 | 36 | 44 | 30 | 30 | 30 | 30 |
| **Sequence Portion** | 1701-1730 | 1721-1750 | 1741-1770 | 1748-1793 | 1779-1814 | 1800-1835 | 1818-1861 | 1848-1877 | 1868-1897 | 1888-1917 | 1908-1937 |
| **Segment index** | **12** | **13** | **14** | **15** | **16** | **17** | **18** | **19** | **20** | **21** | **22** |
| **No. of AA** | 30 | 30 | 30 | 32 | 30 | 30 | 41 | 40 | 38 | 39 | 32 |
| **Sequence Portion** | 1928-1957 | 1948-1977 | 1968-1997 | 1988-2019 | 2008-2037 | 2028-2057 | 2048-2088 | 2063-2102 | 2085-2122 | 2110-2148 | 2130-2161 |
| **Segment index** | **23** | **24** | **25** | **26** | **27** | **28** | **29** | **30** | **31** | **32** | **33** |
| **No. of AA** | 30 | 40 | 35 | 41 | 30 | 30 | 34 | 35 | 30 | 30 | 11 |
| **Sequence Portion** | 2150-2179 | 2163-2202 | 2192-2226 | 2207-2247 | 2233-2262 | 2253-2282 | 2273-2306 | 2293-2327 | 2313-2342 | 2333-2362 | 2353-2363 |
