# Supplementary material for: The Interaction of CRM1 and the Nuclear Pore Protein Tpr
Source: PLoS One. 2014 Apr 10;9(4):e93709. doi: 10.1371/journal.pone.0093709 (PMC3983112; doi:10.1371/journal.pone.0093709)
Supplement: Table S2 — List of the Tpr fragments interacting with each binding site. (DOCX) [file pone.0093709.s006.docx]

Table S2. List of the Tpr fragments interacting with each binding site.

| **Binding Site** | **Tpr Fragment #**  **Sim1** | **Tpr Fragment #**  **Sim2** |
| --- | --- | --- |
| 1 | 18 | 19 |
| 2 | 11 | 7, 27 |
| 3 | 17 | 1 |
| 4 | 16 | 16 |
| 5 | 25 | 4 |
| 6 | 9 | 6, 16 |
| 7 | 6 | 25 |
| 8 | 29 | 31 |
| 9 | 11 | 12 |
| 10 | 30 | 11 |
| 11 | 2, 27 | - |
